# Functional Microdomains in Heart’s Pacemaker: A Step Beyond Classical Electrophysiology and Remodeling

**DOI:** 10.3389/fphys.2018.01686

**Published:** 2018-11-27

**Authors:** Di Lang, Alexey V. Glukhov

**Affiliations:** Department of Medicine, School of Medicine and Public Health, University of Wisconsin-Madison, Madison, WI, United States

**Keywords:** sinoatrial node, pacemaker, microdomain, ion channel, remodeling, signaling complex

## Abstract

Spontaneous beating of the sinoatrial node (SAN), the primary pacemaker of the heart, is initiated, sustained, and regulated by a complex system that integrates ion channels and transporters on the cell membrane surface (often referred to as “membrane clock”) with subcellular calcium handling machinery (by parity of reasoning referred to as an intracellular “Ca^2+^ clock”). Stable, rhythmic beating of the SAN is ensured by a rigorous synchronization between these two clocks highlighted in the coupled-clock system concept of SAN timekeeping. The emerging results demonstrate that such synchronization of the complex pacemaking machinery at the cellular level depends on tightly regulated spatiotemporal signals which are restricted to precise sub-cellular microdomains and associated with discrete clusters of different ion channels, transporters, and regulatory receptors. It has recently become evident that within the microdomains, various proteins form an interacting network and work together as a part of a macromolecular signaling complex. These protein–protein interactions are tightly controlled and regulated by a variety of neurohormonal signaling pathways and the diversity of cellular responses achieved with a limited pool of second messengers is made possible through the organization of essential signal components in particular microdomains. In this review, we highlight the emerging understanding of the functionality of distinct subcellular microdomains in SAN myocytes and their functional role in the accumulation and neurohormonal regulation of proteins involved in cardiac pacemaking. We also demonstrate how changes in scaffolding proteins may lead to microdomain-targeted remodeling and regulation of pacemaker proteins contributing to SAN dysfunction.

## Introduction

The sinoatrial node (SAN) is the primary pacemaker of the heart. Spontaneous beating of the SAN is initiated, sustained, and regulated by a complex system that integrates ion channels and transporters located on the cell membrane surface (often referred to as “membrane clock”) with subcellular calcium handling machinery (by parity of reasoning referred to as an intracellular “Ca-Clock”) (Lakatta and DiFrancesco, 2009; Lakatta et al., 2010). Stable, rhythmic beating of the SAN is ensured by a rigorous synchronization between these two clocks highlighted in the coupled-clock system concept of SAN timekeeping. Following achievement of the maximal diastolic potential, K^+^ current (a combination of a rapidly recovering transient outward current *I*_to_, and a rapidly, *I*_Kr_, and slowly, *I*_Ks_, activating delayed rectifier currents) conductance decreases which unmasks several inward background currents. Together with hyperpolarization-activated current (*I*_f_) and low-voltage activated T-type Ca^2+^ current (*I*_Ca,T_), these start gradual changes of the membrane potential (V_m_) (early diastolic depolarization). Then, spontaneous and rhythmic submembrane local Ca^2+^ releases (LCR) from ryanodine receptors (RyRs) occur and activate an inward Na^+^/Ca^2+^ exchange (NCX) current (*I*_NCX_) to boost diastolic depolarization rate and fire an action potential (AP) via activation of L-type Ca^2+^ current (*I*_Ca,L_).

Until recently, the prevailing concept of cardiac electrophysiology has been that ion channels and receptors are freely mobile in the plasma membrane providing uniform activity through the sarcolemma. Though such simplification was beneficial for computational modeling and enabled the development of relatively straightforward biophysical models based on non-linear dynamics and oscillatory theory, it recently became evident that this simple “random collision model” is inadequate to explain the emerging experimental results which highlight microdomain-specific regulation of cardiomyocyte physiology (reviewed in details elsewhere, Zaccolo and Pozzan, 2002; Warrier et al., 2007; Best and Kamp, 2012; Balycheva et al., 2015; Vinogradova et al., 2018). In the SAN, these include findings on a complex spatial-temporal coupling between the membrane- and Ca^2+^ clocks confirmed in various species, including human (Kim et al., 2018; Tsutsui et al., 2018), synchronization of spontaneous LCRs between discrete RyR clusters (Stern et al., 2014; Torrente et al., 2016), compartmentalized autonomic regulation of pacemaker ion channels which relies on tightly confined cAMP signaling (Barbuti et al., 2004; St Clair et al., 2017; Vinogradova et al., 2018), as well as microdomain-specific remodeling of ion channels secondary to structural alterations including changes in scaffolding proteins (Le Scouarnec et al., 2008; Alcalay et al., 2013; Bryant et al., 2018). The emerging results demonstrate that the functioning of the complex pacemaking machinery at the cellular level depends on tightly regulated spatiotemporal signals which are restricted to precise subcellular microdomains and associated with discrete clusters of different ion channels, transporters and regulatory receptors. Within different subcellular compartments, various proteins form an interacting network and work together as a part of a macromolecular signaling complex. These protein–protein interactions are tightly controlled and regulated by a variety of neurohumoral signaling pathways, and the diversity of cellular responses achieved with a limited pool of second messengers is made possible through the organization of essential signal components in particular microdomains. Importantly, on a tissue level, these are manifested by a dynamic pattern of beat-to-beat migration of leading pacemaker location within the SAN at baseline and during autonomic stimulation, a complex interaction between discrete pacemaker clusters, and the development of SAN arrhythmias associated with pathological remodeling which could not be described in simplified oscillatory models of cardiac pacemaking.

**FIGURE 1:**
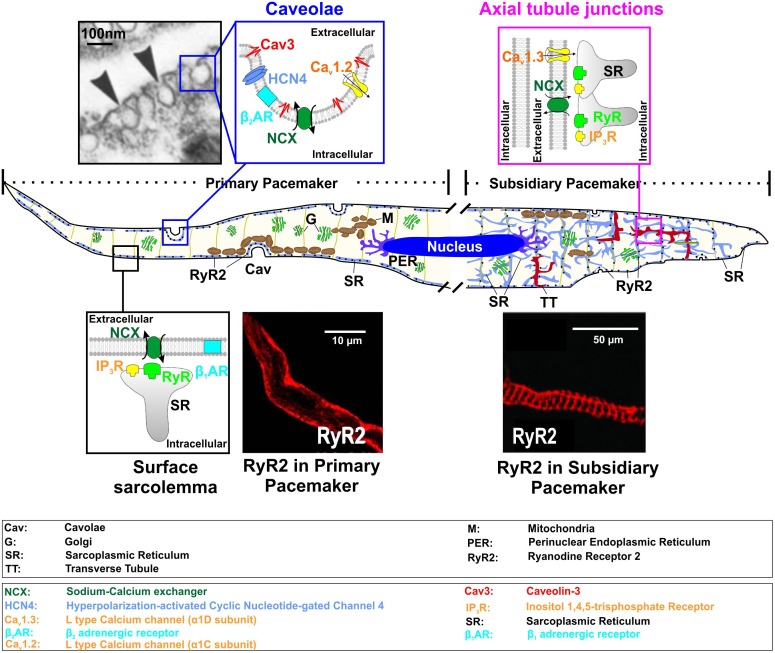
Functional microdomains in SAN myocytes shown for primary *(left part of a model cell)* and subsidiary *(right part)* pacemaker cells, including caveolae [electron microscopy photograph used from (Masson-Pevet et al., 1980) with permission], surface sarcolemma with subsarcolemmal distribution of RyRs (immunofluorescent staining of primary SANCs for RyR2; from Le Scouarnec et al. (2008) with permission), and axial tubule junction both subsarcolemmal and striated distribution of RyRs (immunofluorescent staining of SANCs for RyR2; from Christel et al. (2012) with permission).

In this review, we highlight the emerging understanding of the functionality of distinct subcellular microdomains in SAN cardiomyocytes (SANCs) (Figure 1) and their role in autonomic regulation of cardiac pacemaking. We also demonstrate how changes in scaffolding proteins may lead to microdomain-targeted remodeling and regulation of pacemaker proteins and contribute to SAN dysfunction (SND).

## Functional Macro- and Micro-Architecture of the SAN

The SAN has a highly complex and heterogeneous structure (reviewed in detail elsewhere, Boyett et al., 2000; Fedorov et al., 2012; Csepe et al., 2016). It was shown in humans (Boineau et al., 1988; Li et al., 2017) and dogs (Glukhov et al., 2013) that the SAN consist of several intranodal pacemaker clusters which have different electrophysiological properties, including automaticity robustness and varying response to autonomic stimulation. These were thought to underlie the dynamic pattern of a beat-to-beat shift of the leading pacemaker location within the SAN pacemaker complex during heart rate change under various conditions (Boyett et al., 2000), the development of intranodal conduction blocks, temporally unexcitable areas, SAN micro-reentry and exit block (Glukhov et al., 2013; Li et al., 2017). On the cellular level, the difference between intranodal pacemaker clusters was linked to distinct pacemaker protein expression profiles as well as cellular microarchitecture (Boyett et al., 2000). The latter is critical for pacemaker proteins’ distribution as well as their communication with each other and with subcellular Ca^2+^ clock. Transmission electron microscopy studies by Ayettey and Navaratnam demonstrated that specialized transversal (T)-tubular system is either absent or far less developed in rat SAN than atrial or ventricular myocytes (Ayettey and Navaratnam, 1978). The authors found that in primary SANCs, T-tubules are represented by short and narrow (about 60 nm in diameter versus 105 and 130 nm in atrial and ventricular myocytes, respectively) invaginations of the sarcolemma, which do not usually penetrate sufficiently far to contact the myofibrillae. Instead, SANCs are highly rich with caveolae structures, i.e., muscle-specific caveolin-3 (Cav-3) scaffolding protein containing a subpopulation of lipid rafts, representing small (50–100 nm in diameter) invaginations of the plasma membrane (Figure 1, ‘*caveolae’* microdomain). Caveolae density in rabbit SANCs is 2-times higher than in atrial and 4 to 5-times higher than in ventricular myocytes as estimated from electron microscopy photographs (Masson-Pevet et al., 1980). Through binding to caveolin-scaffolding domain, Cav-3 compartmentalizes and concentrates various proteins, including ion channels, transporters, G-protein subunits, kinases, endothelial nitric oxide synthase (eNOS), and others, many of which contribute to SAN pacemaking.

Through, it is quite difficult to localize the center SAN in electron microscopy studies without functional characterization of the leading pacemaker localization, Ayettey and Navaratnam highlighted the presence of some transitional cells within the SAN region. These transitional myocytes resemble nodal cells in diminutiveness of size and lack of atrial granules and also possess a sparse and disorganized T-tubule system (Ayettey and Navaratnam, 1978). It appears that a sparse tubular system in SANCs is likely different from that in working ventricular myocytes, and may rather represent a ‘super-hub’ of Ca^2+^ signaling associated with axial tubule junctions that rapidly activate Ca^2+^ release through cell-specific molecular microdomain mechanisms and was recently proposed for atrial myocytes by the Lehnart’s group (Brandenburg et al., 2016, 2018). The authors demonstrated that axial tubule junctions in atrial myocytes are highly enriched by cholesterol-rich nanodomains visualized by the fluorescent cholesterol analog dye Chol-PEG-KK114 in live cells (Figure 1, ‘*axial tubule junctions*’), in contrast to ventricular myocytes where T-tubules rarely contain caveolae-shaped membrane structures. Axial tubule junctions form contact junctions with the sarcoplasmic reticulum (SR) and are associated with highly phosphorylated RyRs (Brandenburg et al., 2016) which may play an important role in SANC pacemaking as discussed below (see section “Ca Clock”). Axial tubule junctions and their role in atrial Ca^2+^ signaling have been shown in mouse, rat, rabbit, pig, and human myocytes (Brandenburg et al., 2018).

Such intrinsic structural heterogeneity of the SAN has been recently confirmed on the functional level as well. By performing consecutively measurement of *I*_f_ and *I*_Ca,L_ from the rabbit pacemaker cells isolated from the intercaval region, including the SAN, Monfredi et al. (2018) found their significantly diverse range. Importantly, *I*_f_, but not *I*_Ca,L_ current density was positively related to baseline beating rate. These data correlate well with the distribution of transversal-axial tubule system within the SAN: primary pacemakers with the fastest spontaneous beating rate do not have T-tubules, express the smallest *I*_Ca,L_ which predominantly rely on extratubular LTCCs, and have the highest pacemaker current *I*_f_, while subsidiary SAN pacemakers possess a rudimentary T-tubule network which results in a significant increase of *I*_Ca,L_ and decrease in *I*_f_. Subsequently, two recent reports from Lakatta’s group demonstrated in guinea pig (Kim et al., 2018) and human SANCs (Tsutsui et al., 2018) several populations of cells which show rhythmic pacemaking activity, dysrhythmic firing, and no spontaneous activity (i.e., ‘dormant’ cells). Dysrhythmic and dormant SANCs have smaller and desynchronized LCR activity than rhythmic SANCs; however, in response to sympathetic stimulation, all dysrhythmic cells and a third of dormant SANCs increased their LCR activity and developed automaticity resulting in spontaneous electrical beating (Kim et al., 2018). Whether or not these cells are associated with different pacemaker clusters and responsible for certain ranges of heart rate, remains open to question. These, however, may provide mechanistic basis for dynamic pacemaker location shift within the SAN as it was observed experimentally in intact optically mapped mouse, canine, and human SAN preparations (Fedorov et al., 2010; Glukhov et al., 2010, 2013, 2015b; Li et al., 2017).

In the following sections, we describe microdomain-specific distribution, functioning, and remodeling of the main components of both membrane and Ca^2+^ clocks. Specifically, we focus on how the changes in scaffolding proteins affect functional pacemaker microdomains and results in SND. Though most of these changes are studied in transgenic mouse models, emerging evidence from SND patients harboring similar mutations, which are summarized in the review for available proteins, support the observed results and highlight microdomain-targeted remodeling as a new dimension to cardiovascular disease.

## Membrane Clock

### Pacemaker Channels

The hyperpolarization-activated, cyclic nucleotide-gated (HCN) ion channels are responsible for generating the pacemaker current (funny current, *I*_f_) which is the inward current that contributes to the early stage diastolic depolarization in the SAN (DiFrancesco, 1993). At diastolic potentials, *I*_f_ is predominantly carried by Na^+^ (DiFrancesco, 1993). *I*_f_ is activated by hyperpolarizing voltage steps, with the threshold potential varies from -40 to -60 mV. The voltage dependence of the *I*_f_ activation is influenced by intracellular cyclic adenosine monophosphate (cAMP); a direct binding of cAMP to this channel increases the open probability via a depolarizing shift in the midpoint activation voltage (V_1/2_) (DiFrancesco and Tortora, 1991).

While HCN4 is the most predominant isoform expressed in rodent SANCs (Marionneau et al., 2005), recent reports showed that all three cardiac HCN isoforms (HCN1, HCN2, and HCN4) are highly expressed in the human SAN (Li et al., 2015). Several studies demonstrated that HCN channels localize to caveolae based on the presence of HCN4 in low-density membrane fractions along with Cav-3 as well as the specific interaction of HCN4 with Cav-3 (Barbuti et al., 2007; Ye et al., 2008). Barbuti et al. (2012) reported that all HCN isoforms have a conserved caveolin-binding domain which impact both channel function and trafficking. Disruption of caveolae by reducing membrane cholesterol using methyl-β-cyclodextrin (MβCD) or by expression of dominant negative caveolin mutants alters the gating of HCN channels by shifting the voltage dependence of the activation by approximately 10 mV in the positive direction (Ye et al., 2008). In addition, β_2_-adrenergic receptor (β_2_AR) modulation of HCN4 channel is lost when caveolae are disrupted, which is supported by co-localization of β_2_ARs and HCN4 channels in caveolae (Barbuti et al., 2007).

Recent studies indicate that caveolae-associated β_2_AR-dependent stimulation of HCN4 channels relies on subcellular compartmentalization of cAMP signaling. First, it was found that HCN4 can be phosphorylated by cAMP-dependent protein kinase A (PKA) at the distal C-terminus, in addition to the well-studied cAMP binding to a conserved cyclic nucleotide binding domain in the proximal C-terminus (Liao et al., 2010). Moreover, PKA activity is necessary for cAMP-dependent signaling between β_2_ARs and HCN channels in SANCs; inhibition of PKA with an inhibitory peptide, PKI, significantly reduced the shift in V_1/2_ produced by β_2_ARs stimulation while does not affect a direct stimulation of the channels by exogenous cAMP and Rp-cAMP (an analog than cannot activate PKA) (St Clair et al., 2013). St Clair and colleagues showed that PKA modulation of HCN4 channels depends on distinct cAMP signaling domains created by subcellular localization of cyclic nucleotide phosphodiesterases 3 and 4 (PDE3 and 4) which are responsible for cAMP degradation in the SANCs. The authors demonstrated that PDE4 inhibition in mouse SANCs produced a PKA-independent depolarizing shift in the V_1/2_ of *I*_f_ at rest, likely via a direct binding of elevated cAMP to the channel, but did not remove the requirement for PKA in β_2_AR-to-HCN signaling. In contrast, PDE3 inhibition produced PKA-dependent changes in *I*_f_ both at rest and in response to β_2_AR stimulation (St Clair et al., 2017). Microdomain-specific localization and activity of PDEs in SANCs have been recently reviewed by Vinogradova et al. (2018) and highlight functional importance of local cAMP microdomains with high and low cAMP levels which are involved in local regulation of coupled-clock system components.

### L-Type Ca^2+^ Channels

In the center of the SAN, where the AP upstroke is slow, and little or no Na^+^ current is expressed, *I*_Ca,L_ is principally responsible for the upstroke. Though Ca_v_1.2 represents the major isoform of the L-type Ca^2+^ channel (LTCC) central pore subunit expressed in the heart, SANCs also express Ca_v_1.3 isoform (Zhang et al., 2002). In comparison to Ca_v_1.2 channels which are activated at -40 mV and mainly contribute to AP upstroke, channels formed by Ca_v_1.3 are activated ∼20 mV more negatively than Ca_v_1.2 and thus play an important role in the generation of diastolic depolarization. Knockout of Ca_v_1.3 in mice decreased *I*_Ca,L_ density by 69% and resulted in severe bradycardia and highly erratic pacing rate in the SAN (Mangoni et al., 2003).

**FIGURE 2:**
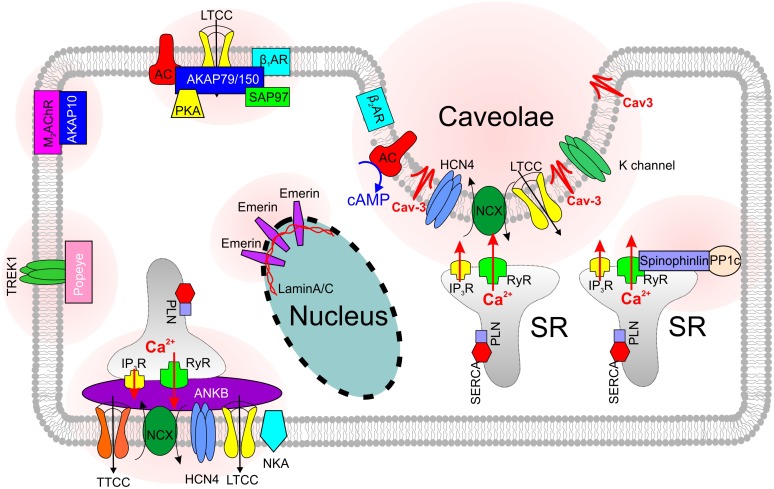
Skeleton of SANC microdomains that are associated with various scaffolding proteins.

A number of important Ca_v_1.2 subpopulations have been identified in cardiomyocytes that associate with unique macromolecular signaling complexes and scaffolding proteins (Figures 1, 2) (Best and Kamp, 2012). These include channels localized to dyadic junctions (i.e., T-tubules) as well as extradyadic channels that reside in biochemically distinct regions of surface membrane, including caveolae, lipid rafts, and plasmalemma. Most of the studies performed on isolated mouse SANCs (Le Scouarnec et al., 2008; Christel et al., 2012) demonstrated a predominant plasma membrane localization of Ca_v_1.2 channels; however, a ratio between caveolar and non-caveolar LTCCs in SANCs is unknown and requires additional studies. In contrast, immunofluorescence analysis of mouse SANCs showed Ca_v_1.3 channels localized both on the plasma membrane (Le Scouarnec et al., 2008) and in sarcomeric structures (presumably, within a sparse T-tubule network observed in subsidiary pacemakers (Ayettey and Navaratnam, 1978) where they co-localize with RyRs (Christel et al., 2012). STimulated Emission Depletion (STED) co-immunofluorescence labeling of mouse atrial myocytes showed that Ca_v_1.3 clusters are located close to Cav-3 clusters in axial tubule junctions where highly PKA phosphorylated clusters of RyR-pS2808 are identified (Brandenburg et al., 2018). Such highly phosphorylated, both at PKA- (P-Ser^2809^) and Ca^2+^/calmodulin-dependent protein kinase II (CaMKII)-dependent (P-Ser^2815^), clusters of RyRs have been found in rabbit SAN and proposed to contribute to spontaneous LCRs. Along with a heterogeneous distribution of T-tubules in the SAN (compare primary vs. subsidiary pacemakers in Figure 1), this may indicate a variable contribution of Ca_v_1.3 to *I*_Ca,L_ in different SAN pacemaker clusters, their functional interaction with RyRs and thus their impact on heart rate. Indeed, Christel and colleagues demonstrated a significantly stronger inactivation of Ca_v_1.2 versus Ca_v_1.3 channels measured in mouse SANCs during strong (+80 mV) depolarization. The authors used a numerical model of mouse SAN automaticity and found that Ca_v_1.3 voltage-dependent facilitation enhanced recovery of pacemaker activity after pauses and positively regulated pacemaking during slow heart rate (Christel et al., 2012). In a subsequent study from the same group, Torrente et al. (2016) showed that Ca_v_1.3 deficiency strongly impaired [Ca^2+^]_i_ dynamics, reducing the frequency of local [Ca^2+^]_i_ release events and preventing their synchronization. These data highlight an importance of microdomain-specific localization of Ca_v_1.3 channels in calcium-voltage clock coupling and in opposing abnormal slowing of heart rate.

Along with distinct functional roles that LTCCs play in cellular microdomains, emerging evidence indicate that subpopulations of LTCCs may possess different biophysical properties resulted from the difference in channel’s structure and/or subcellular microenvironment influence, and therefore experience diverse pathological remodeling. For example, extratubular LTCCs measured in rat and human atrial myocytes, which express both Ca_v_1.2 and Ca_v_1.3, demonstrate low single channel activity (Glukhov et al., 2015a) indicating either a different LTCC structure (both on pore-forming Ca_v_1.2 and Ca_v_1.3 or regulatory Ca_v_β_1-4_ and α_2_δ (Foell et al., 2004) subunits) or microdomain-specific regulation, including the association with various scaffolding proteins. β-subunits enhance channel trafficking to the distinct plasma membrane microdomains, increase the channel’s open probability, cause a hyperpolarization shift in the voltage-dependence of activation, and affect voltage-dependent inactivation (Foell et al., 2004). Though Ca_v_β_2_ is the most abundant β subunit expressed in the heart, both Ca_v_β_1_ and Ca_v_β_3_ mRNA expression have been found in mouse (Marionneau et al., 2005) and rat (Yanni et al., 2011) SAN, and their mRNA levels were higher than those measured in atrial and ventricular myocardium. Similar to Ca_v_β, α_2_δ subunits are also involved in channel’s trafficking via interactions with other proteins, such as extracellular matrix and other membrane proteins involved in cell adhesion (Whittaker and Hynes, 2002).

Along with spatial compartmentalization, LTCCs show a microdomain-specific cAMP- mediated and PKA-dependent regulation, both at baseline and during autonomic stimulation [reviewed in details elsewhere (Best and Kamp, 2012; Vinogradova et al., 2018) and discussed below in ‘Ca^2+^ clock’ section]. The specificity of cAMP signaling is modulated by the binding of PKA to A kinase-anchoring proteins (AKAPs) (Figure 2), which target the kinase to specific intracellular locations and LTCC pools, and provide spatiotemporal control over cAMP signaling events. Various AKAPs serve as a scaffold to assemble proteins involved in Ca^2+^ signaling regulation via microdomain-specific phosphorylation of different subpopulations of LTCCs thus adding another level of complexity to *I*_Ca,L_ and Ca^2+^ signaling regulation (see section “SND and Scaffolding Proteins”).

### T-Type Ca^2+^ Channels

Hagiwara et al. (1988) reported the existence of both long-lasing (*I*_Ca,L_) and transient (*I*_Ca,T_) Ca^2+^ currents in isolated rabbit SANCs. The authors described their distinct biophysical properties, including voltage dependencies for activation/inactivation and pharmacology. Threshold for activation of *I*_Ca,T_ was around -60 mV, and *I*_Ca,T_ was fully activated at about -10 mV. Because activation range of *I*_Ca,T_ overlapped the pacemaker potential of SAN, the potential involvement of *I*_Ca,T_ in the generation of the diastolic depolarization in SANCs was proposed. Indeed, selective inhibition of *I*_Ca,T_ by Ni^2+^ (Hagiwara et al., 1988) or mibefradil (Protas and Robinson, 2000) resulted in significant slowing of spontaneous activity of isolated rabbit SANCs by selective suppression of the later phase of pacemaker depolarization in a concentration-dependent manner, with no effects on AP amplitude and duration.

Two out of three T-type Ca^2+^ channel α-subunits, namely Ca_v_3.1 (α_1H_) and Ca_v_3.2 (α_1I_), were detected in the heart. In contrast to Ca_v_1, Ca_v_3 channels produce large currents in the absence of co-expressed accessory β or α_2_δ subunits, and therefore these proteins are not obligate auxiliary subunits for Ca_v_3 channels. Genetic inactivation of Ca_v_3.1 channels significantly slowed the intrinsic *in vivo* heart rate, prolonged the SAN recovery time, and slowed pacemaker activity of mouse SANCs through a reduction of the slope of the diastolic depolarization (Mangoni et al., 2006). In contrast, Ca_v_3.2-deficient mice displayed normal sinus rhythm without arrhythmias (Chen et al., 2003).

To date, experimental data on T-type Ca^2+^ channels subcellular distribution, including SAN, is limited. In mice with cardiac-specific, conditional expression of the Ca_v_3.1 channels, immunocytochemical labeling revealed their presence primarily on the surface sarcolemma of ventricular myocytes, with less staining within the T-tubules (Jaleel et al., 2008). Osmotic shock, which selectively eliminates T-tubules, induced a greater reduction in L- versus T-type Ca^2+^ currents. Ca^2+^ influx through T-type Ca^2+^ channels also did not induce normal SR Ca^2+^ release which suggests that T- and L-type Ca^2+^ channels are located in different portions of the sarcolemma (Jaleel et al., 2008). Similarly, in wild type mouse atrial myocytes, Curran et al. (2015) showed both Ca_v_3.1 and Ca_v_3.2 expressed on the plasma membrane and, surprisingly, over the atrial Z-line while their association with T-tubules is questionable. Co-immunoprecipitation analysis suggested an association of Ca_v_3.1 and Ca_v_3.2 channel isoforms with caveolae scaffolding protein Cav-3 in neonatal mouse ventricular myocytes (Markandeya et al., 2011). Interestingly, co-expression of Cav-3 significantly decreased the peak Ca_v_3.2 current density in HEK293 cells, whereas co-expression of Cav-3 did not alter peak Ca_v_3.1 current density. It is possible that Ca_v_3.1 and Ca_v_3.2 channels are located on the surface sarcolemma, in association with different structural microdomains (Figure 1).

### Na^+^/Ca^2+^ Exchanger

NCX plays a critical role in SAN pacemaking by producing a depolarizing current and boosting depolarization rate in late diastole when local Ca^2+^ released by RyRs beneath the cell surface membrane (Figure 1, *primary pacemaker*) (Lakatta et al., 2010). NCX1 is the predominant isoform expressed in the heart. Atrial-specific NCX knock-out in mice resulted in severe pacemaker abnormalities manifested by “tachy-brady” arrhythmias and associated with quiescent isolated SANCs where, however, copious intracellular Ca^2+^ waves were present but failed to trigger APs (Torrente et al., 2015). These highlight a key role of NCX in synchronizing Ca^2+^ and membrane clocks.

Though data on NCX distribution in SANCs is limited, studies on atrial and ventricular myocytes indicate that NCX membrane localization might be associated with different targeting components. Immunohistological evidence from rabbit SANCs indicate significant submembrane co-localization of NCX1 and RyRs which exceeds that measured in atrial and ventricular myocytes (Lyashkov et al., 2007) where NCX1 labeling is present throughout the cell (attribute to an extensive T-tubular network) (compare ‘*caveolae’* vs. ‘*axial tubule junction’* compartments in Figure 1). Such localization allows crosstalk between RyR LCRs and NCX (Figure 1, ‘*surface sarcolemma’* compartment). Several groups have identified NCX associated with Cav-3 by both co-immunoprecipitation and immunolabeling (Scriven et al., 2005; Camors et al., 2006) with the latter finding those complexes enriched on the cell surface and little in the T-tubules (Scriven et al., 2005). It was also reported that NCX1 has several caveolin binding motifs and that the NCX1 protein associates with Cav-3 (Bossuyt et al., 2002). Others, using the same techniques, have found little, if any, association (Cavalli et al., 2007).

### Other Channels

Besides the ion channels discussed above, there are other ion channels that participate in the SAN pacemaking. These include (1) ion channels involved in store-operated Ca^2+^ entry (SOCE); (2) SR-Ca^2+^ activated non-selective Na^+^ transient receptor potential melastatin 4 (TRPM4) ion channel; (3) sodium channels Na_v_1.5 which are likely expressed the periphery but not in the center of the SAN; and (4) chloride channels. All these channels provide an additional inward current during spontaneous diastolic depolarization and thus may contribute to SAN pacemaking. Though these channels may not play a prominent role in SAN activity, the mutations of the genes that encode them are reported to associate with SND observed clinically, suggesting their contributions to the SAN pacemaking. Importantly, most of those channels are associated with different structural proteins and have been linked to distinct microdomains, including caveolae/lipid rafts, intercalated disks, luminal SR, etc., and thus may require a precise spatial arrangement to support their functional coupling and integrity. Disruption in one of the protein localization could affect other proteins involved in a complex protein–protein interaction and thus disturb pacemaker automaticity. Finally, potassium ion channels, including a rapidly recovering transient outward current *I*_to_ (K_v_4.2), a rapidly, *I*_Kr_ (ERG), and slowly activating, *I*_Ks_ (K_v_LQT1), delayed rectifier currents, acetylcholine-activated K^+^ current *I*_K,ACh_ (K_ir_3.1 and K_ir_3.4), and ATP-sensitive K^+^ current *I*_K,ATP_ (K_ir_6.2), are expressed in the SAN and also demonstrate microdomain-specific distribution and regulation, as reviewed in details elsewhere (Balycheva et al., 2015).

## Ca-Clock

Besides ion channels that depolarize SAN V_m_ during diastole, spontaneous Ca^2+^ oscillations also play a critical role in pacemaking (Lakatta et al., 2010). Attributed to the ‘Ca-clock’ component of the pacemaking system, this [Ca^2+^]_i_ cycling activity is comprised of spontaneous local Ca^2+^ releases from the RyRs (i.e., AP-independent Ca^2+^ releases, or LCRs, in contrast to AP-triggered SR Ca^2+^ releases, or Ca^2+^ transients, CaT) and Ca^2+^ reuptake back to the SR via SERCA. Emerging evidence shows that disruption of LCR activity leads to dramatic changes in SAN pacemaking (Vinogradova and Lakatta, 2009; Gao et al., 2010). LCRs occur during the late phase of the diastolic depolarization. They generate small increments in the local [Ca^2+^]_i_ which activates NCX to pump Ca^2+^ out of the cell in exchange for Na^+^ ions at the ratio of 1 Ca^2+^: 3 Na^+^, therefore leading to a net positive charge influx and a subsequent depolarization of the V_m_ to the threshold of the next beat. Unlike AP-triggered CaT, LCRs are V_m_-independent, rhythmic activities that are localized near the sarcolemmal membrane (Vinogradova et al., 2004). Such highly localized LCR patterns are associated with a unique distribution of RyR found in SANCs. In contrast to atrial and ventricular myocytes, immunofluorescent staining studies performed on SANCs demonstrated that RyRs are exclusively expressed beneath the sarcolemma in rabbit (Musa et al., 2002), guinea-pig (Rigg et al., 2000) and mouse (Le Scouarnec et al., 2008) SANC from a central SAN area. Internal labeling of RyRs that followed a striation pattern was also observed by other groups in mouse and guinea-pig SANs (Rigg et al., 2000; Christel et al., 2012) as well as in rabbit myocytes isolated from a peripheral SAN area (Musa et al., 2002) (compare immunofluorescent staining images for RyR2 distribution in primary and subsidiary pacemakers in Figure 1).

Molecular mechanisms that underlie the generation and regulation of LCR activity, are still incompletely understood. Multiple factors have been proposed to contribute to these spontaneous SR Ca^2+^ releases including an elevated SR Ca^2+^ load (i.e., [Ca^2+^]_SR_) as well as hyperactivity of RyRs due to their hyperphosphorylation or hypersensitization via IP_3_R-mediated Ca^2+^ release.

SR Ca^2+^ release depends on the level of [Ca^2+^]_SR_ which affects the sensitivity of RyR2 (Bassani et al., 1995). Though no accurate [Ca^2+^]_SR_ was reported in SANCs, atrial myocytes possess a threefold higher SR Ca^2+^ load compared to ventricular myocytes (Walden et al., 2009). Though amplitude of caffeine-evoked CaT in rabbit central SANCs is ∼50% lower than that in atrial myocytes (Jones et al., 2008), SANCs have a dramatically smaller size than atrial myocytes and thus a higher single SR Ca^2+^ load might be expected in SANCs. Such “Ca^2+^ overload” may lead to spontaneous Ca^2+^ releases such as LCRs. “Ca^2+^ overload” then gets restored after Ca^2+^ is replenished by SERCA from AP-induced CaT, thus the next leak follows and rhythmic LCRs are generated.

SR Ca^2+^ load is tightly associated with a SR protein calsequestrin (Casq2). Casq2 is a low-affinity, high-capacity Ca^2+^ binding protein expressed in cardiomyocytes (Knollmann et al., 2006). Overexpression of Casq2 in transgenic mice (Jones et al., 1998) causes a significant increase in the SR Ca^2+^ load and SR Ca^2+^ release. With such increase, a higher heart rate was indeed observed in mice overexpressing *Casq2* (Jones et al., 1998). Accordingly, bradycardia was reported in *Casq2* knockout mice (Glukhov et al., 2015b).

IP_3_Rs may also contribute to the LCR generation via hypersensitization of RyRs. IP_3_Rs are another type of SR Ca^2+^ releasing channels which are activated by IP_3_ through the hydrolysis of phosphatidylinositol-(4,5)-bisphosphate by phospholipase C. Recent studies demonstrated that this process might be confined within different microdomains including lipid rafts (Delos Santos et al., 2015) and dorsal ruffles (Gu and Neel, 2003). Stimulation of IP_3_Rs was found to accelerate a spontaneous beating rate of mouse SANCs likely through regulation of Ca^2+^ spark parameters and RyR open probability (Ju et al., 2011). In heart failure rabbit atrial myocytes, upregulation of IP_3_R-induced Ca^2+^ releases was detected and linked to the enhanced spontaneous SR Ca^2+^ releases (Hohendanner et al., 2015). Unlike RyRs, IP_3_Rs seem to express both beneath the sarcolemma membrane and inside SANCs (Figure 1), with the ones near membrane found partially co-localized with HCN4, RyR, and SERCA (Ju et al., 2011). In canine ventricular myocytes, a direct interplay between IP_3_-mediated G_q_ protein coupled receptor signaling pathway and Cav-3 has been demonstrated (Guo et al., 2011), linking IP_3_-dependent non-junctional Ca^2+^ activities to subsarcolemmal caveolae. Significant decrease in the occurrence of spontaneous Ca^2+^ sparks was reported in rat and human atrial myocytes treated with MβCD, which depletes cholesterol and destroys caveolae structures (Glukhov et al., 2015a). Stimulation of IP_3_Rs through sphingosine kinase 1 is shown to control the Ca^2+^ release in caveolar microdomains (Pulli et al., 2015). IP_3_Rs were also associated with structural protein AnkB: their expression and membrane targeting were found to be significantly reduced in AnkB^+/-^ mice (Le Scouarnec et al., 2008).

Phosphorylation of RyRs, SERCA and phospholamban (PLN), are also suggested to regulate LCRs. cAMP-mediated, PKA- (Vinogradova et al., 2006) and CaMKII-dependent phosphorylation (Vinogradova et al., 2000; Li et al., 2016) of RyRs have been reported to affect the size and rhythm of LCRs in rabbit SANCs. At the same time, knock-in alanine replacement of RyR phosphorylation sites PKA (S2808) or CaMKII (S2814) did not affect heart rate responses to isoproterenol *in vivo* or in isolated SANCs in mice (Wu et al., 2016). Furthermore, Wu et al. (2016) demonstrated that selective mutations of PLN phosphorylation sites PKA (S16) or CaMKII (T17) in mice also did not affect heart rate. Therefore, although phosphorylation of SR Ca^2+^ proteins may contribute in SAN LCRs, they appear to not affect heart rate by one single target site governing SR Ca^2+^ uptake or release. Recent studies found that RyR phosphorylation follows a highly localized pattern and was linked to specific subcellular microdomains including lipid rafts (Younes et al., 2008; Lukyanenko et al., 2016).

## cAMP

Although cardiac pacemaking, at rest and during the sympathetic fight-or-flight response, was shown to depend on cAMP signaling in SAN myocytes, cAMP does not directly regulate Ca-clock and SR Ca^2+^ cycling (Lakatta et al., 2010). Instead, it regulates the membrane clock functioning via a direct binding to HCN4 channels. In addition, cAMP modulates the Ca-clock via the regulation of PKA-dependent phosphorylation of SR proteins including RyR, SERCA and PLN. SANCs have a high basal level of cAMP due to a high constitutive activation of adenylyl cyclase (AC), the enzyme that converts adenosine triphosphate to cAMP (Vinogradova et al., 2006). Such high basal level of cAMP may facilitate the periodical LCR activities in the SAN.

With a progress in the development of biosensor techniques, different pools of cAMP activities between the plasma membrane and the bulk cytoplasmic compartment have been observed. Recent studies unveiled that cAMP activity may be more compartmentalized within discrete subcellular microdomains, including lipid rafts and caveolae (Younes et al., 2008). It was shown that activities of some ACs (Younes et al., 2008) and PDEs (Senzaki et al., 2001) that are responsible for cAMP synthesis and degradation, respectively, are restricted to lipid rafts in SANCs. In the SAN, high basal PDE activity (Vinogradova et al., 2008; Hua et al., 2012) is one of the control checkpoints. Inhibition of basal PDEs in SANCs markedly elevates cAMP-mediated phosphorylation of membrane and Ca-clocks’ proteins, resulting in an acceleration of spontaneous AP firing rate in the rabbit SAN (Vinogradova et al., 2008). PDE2-4 are the most abundant in the mouse SAN (Hua et al., 2012) where they regulate the beating rate of atrial preparations (Galindo-Tovar and Kaumann, 2008), AP firing rate, and *I*_Ca_ in SANCs (Hua et al., 2012). In rat ventricular myocytes, inhibition of PDE4, similar to MβCD treatment, results in loss of confined βAR-mediated cAMP signaling, resulting in a cell-wide cAMP signal propagation patterns (Nikolaev et al., 2010). In SANCs, caveolae may localize cAMP signaling to confined subsarcolemmal microdomains, similar to that observed in ventricular myocytes, and thus participate in compartmentalized regulation of both clocks. Downregulation of caveolae scaffolding protein Cav-3 in failing ventricular myocytes (Nikolaev et al., 2010) as well as expression of the dominant negative Cav-3 mutant (Wright et al., 2014), or disruption of caveolae by MβCD (Calaghan et al., 2008), converts the sarcolemmal-confined cAMP signal to a global signal that targets proteins of the SR and myofilaments, increasing cell contractility and CaT amplitude. This was not linked to the modulation of *I*_Ca,L_, but instead to a discrete PKA-mediated phosphorylation of PLN (Macdougall et al., 2012). Recently, by using a PLN-linked cAMP biosensor, Sprenger et al. (2015) uncovered the existence of compartmentalized SERCA phosphorylation mediated by cAMP in adult mouse ventricular myocytes. Because SERCA activity is critical in the regulation of [Ca^2+^]_i_ balance and SR Ca^2+^ cycling in SANCs (Logantha et al., 2016), one could expect that this compartmentalized phosphorylation may be important for the determination of distribution and size of LCRs in the regulation of the Ca-clock.

## CaMKII

Ca^2+^/calmodulin-dependent protein kinase II (CaMKII) is another important component regulating the coupled-clock system. CaMKII is involved in the regulation of both clocks and participates in both physiological (Vinogradova et al., 2000; Li et al., 2016) and pathological (Luo et al., 2013) activities of the SAN. CaMKII is a Ser/Thr protein kinase and is known as one of the major downstream targets of Ca^2+^ signaling. In SANCs, CaMKII senses subcellular Ca^2+^ changes and is activated via binding to Ca^2+^-calmodulin (CaM) complex at the CaM regulatory domain (Wu and Anderson, 2014). Activated CaMKII catalyzes phosphorylation of both L- and T-type Ca^2+^ channels, PLN (Grimm and Brown, 2010) and RyRs (Wehrens et al., 2004).

Recent studies suggested that CaMKII activity may be confined to some specific membrane microdomains. Caveolae-specific activation of CaMKII was also detected in cardiomyocytes and linked to caveolae-localized phosphorylation of LTCCs (Tonegawa et al., 2017). In ventricular myocytes, a local Ca^2+^/reactive oxygen species (ROS) function microdomain was reported, where a cluster of RyRs directly couples to CaMKII and ROS and thus gets a direct modulation from local CaMKII activity (Dries et al., 2013). In such localized microdomains, CaMKII is found to translocate to specific target compartments, and spatial barriers to CaMKII phosphorylation could be overcome by its translocation and anchoring to the substrate itself or to nearby target protein within the localized compartments (Tsui et al., 2005).

These findings highlight a critical role of localized CaMKII-mediated regulation of Ca^2+^ handling proteins and suggest a potential role of microdomain-specific activity of CaMKII in the regulation of SAN pacemaking. Furthermore, CaMKII- and cAMP-mediated regulation are reported to share microdomain location in SAN myocytes where a cross talk between CaMKII and PDE1 is found (Lukyanenko et al., 2016). Deciphering the disruptions of such localized regulation in SAN may create a new direction for SND treatment in the future.

## Compartmentalized Autonomic Regulation in SANCs

### Compartmentalized β-Adrenergic Receptors

β_1_- and β_2_ARs are the primary sympathetic receptors in the heart and play different roles in the regulation of cardiac rhythm. In early 2000s, researchers already found that both ARs show a compartmentalized distribution in cardiomyocytes: β_2_ARs are predominantly concentrated in T-tubules and caveolar structures, whereas β_1_ARs are mainly localized in the non-caveolar membrane and non-lipid raft heavy fractions of plasma membrane (Rybin et al., 2000) (Figure 1). Distinct functional domains then were found in cardiomyocytes to conduct β_1_- and β_2_-adrenergic signaling (Shcherbakova et al., 2007). The cardiomyocyte membrane was reported to develop into specialized zones associated with scaffold proteins SAP97 and AKAP79/150, where β_1_ARs are found enriched, whereas, β_2_ARs are excluded from such microdomains (Shcherbakova et al., 2007) (Figure 2). Disrupted compartmentalized pattern of βARs was shown to be involved in ventricular myocyte remodeling associated with heart failure (Nikolaev et al., 2010).

In SAN myocytes, β_2_/β_1_ expression ratio is higher comparing to atrial and ventricular myocytes (Brodde et al., 1982), and a higher efficiency of β_2_ARs was observed (Brodde et al., 2001). Co-immunoprecipitation and immunocytochemistry studies found in rabbit SANCs that β_2_ARs co-localized with HCN4 channels in the caveolar microdomain (Figure 1), and their disruption via cholesterol depletion by MβCD completely abolished the effect of β_2_-adrenergic stimulation, while the effect of β_1_-adrenergic stimulation still maintained (Barbuti et al., 2007). Altogether, these suggest that the β-adrenergic stimulation in the SAN is presented in a compartmentalized pattern, in which, β_1_- and β_2_ARs localize and function in distinct microdomains.

### Compartmentalized Muscarinic Receptors

M_2_ muscarinic receptors are the primary type of muscarinic receptors expressed in cardiac myocytes. It was shown that M_2_ receptors are located outside of caveolar fractions of plasma membrane (Feron et al., 1997). Upon parasympathetic stimulation, the translocation of M_2_ receptors to caveolar fractions was detected using a PKA FRET-based biosensor (Warrier et al., 2005). It is reported that the M_2_ receptor-mediated cAMP response is associated with distinct AC isoforms expressed in different membrane microdomains (Iancu et al., 2007).

## SND and Scaffolding Proteins

Etiology of SND may include both intrinsic and extrinsic reasons. Prevailing intrinsic factors leading to SND are associated with mutations or dysfunctions of key components in the coupled clock systems including HCN4 channel, potassium channel (*KCNQ1*), sodium channel (*SCN5A*), RyR and others. Emerging evidence has shown that dysfunction of structural or scaffolding proteins could also result in SND (Table 1). Below, we summarize several scaffolding proteins which dysfunctions could lead to SND.

**Table 1:** SAN pacemaking abnormalities linked to mutations in scaffolding proteins and associated remodeling of the coupled-clock pacemaking system.

Protein	Gene	Species	Condition	Dysfunction	Associated pacemaking component remodeling		Reference
						
					Membrane clock	Calcium clock	
AKAP10	*AKAP10*	Human	646V	Fast HR; low HRV	AChR-mediated targets	AChR-mediated targets	Tingley et al., 2007
		Mouse	Global I646V	Bradycardia; sinus pauses	AChR-mediated targets	AChR-mediated targets	Tingley et al., 2007
Ankyrin-B	*ANK2*	Human	E1425G	Bradycardia; AF	NCX; NKA	IP_3_R	Mohler et al., 2003; Le Scouarnec et al., 2008
		Mouse	Global AnkB^+/-^	Bradycardia; high HRV	NCX(↓I_NCX_); NKA; Cav1.3 (↓l_CaL_)	IP_3_R	Le Scouarnec et al., 2008
Caveolin-3	*CAV3*	Human	T78M	Bradycardia; tarchycardia; AF	Kv1.5; HCN4	cAMP signaling	Campostrini et al., 2017
Emerin	*EMD*	Human	Lys37del	Bradycardia; AF	?	?	Karst et al., 2008
Lamin A/C	*LMNA*	Human	A331G	Bradycardia; AF	?	?	Hoorntje et al., 2017
MHC-α	*MYH6*	Human	A721T	Sick sinus syndrome	?	?	Holm et al., 2011
Popeye	*POPDC1.2*	Mouse	Global Popdd, 2^-/-^	Bradycardia; sinus pauses	*I_K_, I_Na_*		Froese et al., 2012
Spinophilin/neurabin	*PPP1R9B*	Mouse	Global Sp^-/-^	Enhanced bradycardiac response to a-adrenergic stimulation	cAMP-mediated targets	RyR; cAMP signaling	Lu et al., 2010; Chiang et al., 2014


*HR: Heart Rate; HRV: Heart Rate Variability*

### Ankyrin-B

Ankyrin-B (AnkB) is an important multifunctional scaffolding protein that is essential for membrane structure organization as well as trafficking and localization of various pacemaker proteins. AnkB syndrome, i.e., a type 4 long QT syndrome, is a rare cardiac arrhythmia syndrome, which is associated with a loss-of-function mutation of AnkB in the heart (Mohler et al., 2004). Dysfunctions in AnkB result in SND in human (Le Scouarnec et al., 2008) and mice (Le Scouarnec et al., 2008; Glukhov et al., 2010) and are associated with atrial fibrillation (Le Scouarnec et al., 2008; Cunha et al., 2011).

Heterozygous knocking out AnkB in mice results in significantly reduced expression of NCX, NKA and IP_3_Rs in the SAN (Figure 2); furthermore, AnkB^+/-^ SANCs show abnormal localization of Ca_v_1.3 and NCX proteins. In contrast to the homogenous membrane distribution of Ca_v_1.3 channels in wild type SANCs, Ca_v_1.3 expression in AnkB^+/-^ cells was limited to an internal perinuclear distribution (Le Scouarnec et al., 2008). This was associated with a concomitant decrease in a whole-cell *I*_Ca,L_ density. Cunha et al. (2011) demonstrated that AnkB directly associates with Ca_v_1.3, and this interaction is regulated by a short, highly-conserved motif on the C4 region of the C-terminus of Ca_v_1.3. Importantly, no changes in Ca_v_1.2 expression and localization were found in AnkB^+/-^ SAN and atrial myocytes. Similar, AnkB^+/-^ mice showed significant reduction of *I*_NCX_ current density in SANCs (Le Scouarnec et al., 2008). Altogether, these resulted in SAN pacemaking abnormalities observed both in single SANCs (Le Scouarnec et al., 2008) and isolated SAN preparations (Glukhov et al., 2010).

### Caveolin-3

Cav-3, the integral membrane protein that is essential in formation of caveolae (Figures 1, 2), is also linked with SND (Lang et al., 2016; Campostrini et al., 2017). As discussed in the previous sections, Cav-3 is involved in the regulation of multiple ion channels and transporters, including those involved in pacemaking. Cav-3 F97C and S141R mutations have been linked to the long QT type 9 inherited arrhythmia syndrome (LQT9) causing AP duration prolongation which had been attributed to an increase in late Na^+^ current (Vatta et al., 2006). In spontaneously beating neonatal cardiomyocytes, the expression of the T78M mutation in *CAV3* gene significantly increased AP peak-to-peak variability without altering neither the mean rate nor the maximum diastolic potential (Campostrini et al., 2017) and was associated with a positive shift of activation of HCN4 channels, in a dominant way. The authors also identified a small cohort of patients with supraventricular arrhythmias including sinus tachycardia, bradycardia, and atrial fibrillation where the T78M Cav-3 variant is more frequent than in the general population. Recent preliminary findings from out laboratory indicate that cardiac-specific conditional knock-out of Cav-3 in mice results in significant beat-to-beat heart rate lability linked with suppressed SAN function, enhanced atrial ectopy and paroxysms of alternating periods of tachycardia-bradycardia rhythm (Lang et al., 2016). These results highlight the importance of Cav-3 in supporting functional integrity of the SAN pacemaker complex.

### Spinophilin

Another scaffolding protein involved in the SAN pacemaking and dysfunction is spinophilin. Spinophilin is ubiquitously expressed (Figure 2) and interacts with a variety of target proteins essential for Ca^2+^ homeostasis and cellular contraction in adult ventricular myocytes (Petzhold et al., 2011). Spinophilin mediates the targeting of protein phosphatase 1 to RyR (Ragusa et al., 2010). Single RyR channel’s open probability was observed to increase in spinophilin knockout mice (Chiang et al., 2014). A recent study reported a bradycardic response to α_2_-adrenergic stimulation found in spinophilin null mice; however, no significant heart rate changes comparing to wild type mice were observed at baseline condition (Lu et al., 2010).

### Popdc Protein

The Popeye domain containing (Popdc, *POPDC1-3*) gene family displays preferential expression in skeletal muscle and the heart, and encode membrane proteins harboring an evolutionarily conserved Popeye domain, which functions as a binding domain for cAMP. Popdc proteins are abundantly present in intercalated disks, lateral membranes and T-tubules (Figure 2). In the heart, atrial expression of Popdc1 is higher than in the ventricle, and the entire cardiac conduction system, including the SAN, displays the most intense expression levels (Froese et al., 2012). Null mutations of members of the Popdc gene family in mice are associated with a stress-induced sinus bradycardia and prominent sinus pauses. Moreover, the phenotype develops in an age-dependent manner, being absent in the young animal and becoming increasingly severe, as the animals grow older. In addition to cAMP binding site, Popeye domain of POPDC1 has the binding sites of KCNK2 (TREK-1, a member of two-pore K^+^ channels family) (Froese et al., 2012) and Cav-3 (Alcalay et al., 2013) proteins. It was found that TREK-1 current was increased twofold in the presence of Popdc1-3 proteins. Along with a prevalent SAN phenotype characterized by bradycardia with frequent episodes of sinus pause following stress in cardiac-specific TREK-1-deficient mice (Unudurthi et al., 2016), this may contribute to SND observed in Popdc-null mice. Finally, Popdc1-null cardiomyocytes showed a statistically significant 70% reduction in caveolae number (Alcalay et al., 2013) which can additionally contribute to SND phenotype via modulation of various pacemaker ion channels in transporters discussed in previous sections.

### A-Kinase Anchor Proteins

Another scaffolding protein family linked to SND, is AKAPs. AKAPs localize PKA to a different subcellular compartment, permitting a higher degree of selectivity and specificity of phosphorylation for different downstream PKA substrates. More than 14 different AKAPs have been shown to be expressed in both rodent and human heart tissue including: AKAP5 (AKAP150/79), AKAP7 (AKAP 15/18), gravin (AKAP12), AKAP9 (yotiao) and mAKAP. Though AKAPs’ function in the SAN is not clear, those proteins are critically important for [Ca^2+^]_i_ regulation and thus may contribute to Ca-clock regulation and SAN pacemaking.

Throughout all AKAPs, AKAP150/79 (AKAP5) is probably the most studied in the heart. AKAP150/79 targets PKA and phosphatases to regions near Ca_v_1.2 (Figure 2) increasing the probability of long openings and coupled gating events between channels. Enzymes known to associate with AKAP150/79 include PKA, protein kinase C, CaM, AC5/6, and PP2B. Sympathetic stimulation of adult cardiomyocytes requires association of AKAP150/79 with a subpopulation of LTCCs coupled with Cav-3 (Nichols et al., 2010). Ablation of AKAP150 in mice with long QT syndrome 8 (LQT8), a disease also known as Timothy syndrome characterized by sinus bradycardia, prolonged QT interval and lethal arrhythmias, restores normal gating in Ca_v_1.2-LQT8 channels and protects the heart from arrhythmias (Cheng et al., 2011). Dysfunction in AKAP10 has been associated with SAN pacemaking and SND in both mice and humans by effecting vagal regulation of the heart (Tingley et al., 2007).

### Other Structural Proteins

A rare variant in MYH6, which encodes protein myosin heavy chain α isoform (MHC-α) is also reported associated with high risk of sick sinus syndrome (Holm et al., 2011). There are also some nuclear structural proteins that have been reported to be involved in the SND. A type II integral membrane protein that anchored to the inner nuclear membrane, emerin (Figure 2), whose mutation is known to develop Emery-Dreifuss Muscular Dystrophy (Emery and Dreifuss, 1966), is reported to associate with SND with underlying mechanism yet to illustrate (Karst et al., 2008). Mutations in the lamin A/C gene LMNA are reported to cause a variety of heart diseases including SND (Figure 2) (Hoorntje et al., 2017). Though the direct mechanisms of nucleus-associated scaffolding protein-induced modulation of SAN activity are unknown, it might be linked to pacemaker protein expression and/or trafficking.

## Summary

Contemporary evidence clearly demonstrates an emerging role of compartmentalized, i.e., associated with distinct, spatially-confined microdomains, organization of pacemaker signaling complexes in the SANCs. Disruption in subcellular targeting of pacemaker proteins and associated signaling molecules upon structural remodeling of the SAN, may affect their biophysical properties and neurohormonal regulation as well as protein–protein interactions within the pacemaker signaling complex disturbing rhythmic generation of APs and thus contributing to the pathophysiology of the SND. These are clear from patients and animal models with genetic defects of scaffolding proteins which are closely associated with SND via the indirect changes of key components in the coupled-clock systems in terms of protein expression, functioning and membrane localization. This extends beyond the classical concept of electrical remodeling, according to which dysfunction can be explained by straightforward increases or decreases in protein expression alone, and adds a new dimension to cardiovascular disease. It thus introduces a novel framework for therapeutic approaches for pacemaker dysfunction treatment targeted at preventing the degradation of cardiac cytoarchitecture.

## Author Contributions

AG and DL substantially contributed to the conception and design of the work; the acquisition, analysis or interpretation of the data and literature; drafting the work critically for important intellectual content; provide approval for publication of the content; agree to be accountable for all aspects of the work in ensuring that questions related to the accuracy or integrity of any part of the work are appropriately investigated and resolved.

## Conflict of Interest Statement

The authors declare that the research was conducted in the absence of any commercial or financial relationships that could be construed as a potential conflict of interest.

## References

[B1] Alcalay Y. Hochhauser E. Kliminski V. Dick J. Zahalka M. A. Parnes D. (2013). Popeye domain containing 1 (Popdc1/Bves) is a caveolae-associated protein involved in ischemia tolerance. *PLoS One* 8:e71100. 10.1371/journal.pone.0071100 24066022 PMC3774711

[B2] Ayettey A. S. Navaratnam V. (1978). The T-tubule system in the specialized and general myocardium of the rat. *J. Anat.* 127(Pt 1), 125–140. 701190 PMC1235649

[B3] Balycheva M. Faggian G. Glukhov A. V. Gorelik J. (2015). Microdomain-specific localization of functional ion channels in cardiomyocytes: an emerging concept of local regulation and remodelling. *Biophys. Rev.* 7 43–62. 10.1007/s12551-014-0159-x 28509981 PMC5425752

[B4] Barbuti A. Gravante B. Riolfo M. Milanesi R. Terragni B. DiFrancesco D. (2004). Localization of pacemaker channels in lipid rafts regulates channel kinetics. *Circ. Res.* 94 1325–1331. 10.1161/01.RES.0000127621.54132.AE 15073040

[B5] Barbuti A. Scavone A. Mazzocchi N. Terragni B. Baruscotti M. Difrancesco D. (2012). A caveolin-binding domain in the HCN4 channels mediates functional interaction with caveolin proteins. *J. Mol. Cell. Cardiol.* 53 187–195. 10.1016/j.yjmcc.2012.05.013 22659290

[B6] Barbuti A. Terragni B. Brioschi C. DiFrancesco D. (2007). Localization of f-channels to caveolae mediates specific beta2-adrenergic receptor modulation of rate in sinoatrial myocytes. *J. Mol. Cell. Cardiol.* 42 71–78. 10.1016/j.yjmcc.2006.09.018 17070839

[B7] Bassani J. W. Yuan W. Bers D. M. (1995). Fractional SR Ca release is regulated by trigger Ca and SR Ca content in cardiac myocytes. *Am. J. Physiol.* 268(5 Pt 1), C1313–C1319. 10.1152/ajpcell.1995.268.5.C1313 7762626

[B8] Best J. M. Kamp T. J. (2012). Different subcellular populations of L-type Ca^2+^ channels exhibit unique regulation and functional roles in cardiomyocytes. *J. Mol. Cell. Cardiol.* 52 376–387. 10.1016/j.yjmcc.2011.08.014 21888911 PMC3264751

[B9] Boineau J. P. Canavan T. E. Schuessler R. B. Cain M. E. Corr P. B. Cox J. L. (1988). Demonstration of a widely distributed atrial pacemaker complex in the human heart. *Circulation* 77 1221–1237. 10.1161/01.CIR.77.6.1221 3370764

[B10] Bossuyt J. Taylor B. E. James-Kracke M. Hale C. C. (2002). The cardiac sodium-calcium exchanger associates with caveolin-3. *Ann. N. Y. Acad. Sci.* 976 197–204. 10.1111/j.1749-6632.2002.tb04741.x 12502561

[B11] Boyett M. R. Honjo H. Kodama I. (2000). The sinoatrial node, a heterogeneous pacemaker structure. *Cardiovasc. Res.* 47 658–687. 10.1016/S0008-6363(00)00135-8 10974216

[B12] Brandenburg S. Kohl T. Williams G. S. Gusev K. Wagner E. Rog-Zielinska E. A. (2016). Axial tubule junctions control rapid calcium signaling in atria. *J. Clin. Invest.* 126 3999–4015. 10.1172/JCI88241 27643434 PMC5096811

[B13] Brandenburg S. Pawlowitz J. Fakuade F. E. Kownatzki-Danger D. Kohl T. Mitronova G. (2018). Axial tubule junctions activate atrial Ca^2+^ release across species. *Front. Physiol.* 9:1227 10.3389/fphys.2018.01227 PMC6187065 30349482

[B14] Brodde O. E. Bruck H. Leineweber K. Seyfarth T. (2001). Presence, distribution and physiological function of adrenergic and muscarinic receptor subtypes in the human heart. *Basic Res. Cardiol.* 96 528–538. 10.1007/s003950170003 11770070

[B15] Brodde O. E. Leifert F. J. Krehl H. J. (1982). Coexistence of beta 1- and beta 2-adrenoceptors in the rabbit heart: quantitative analysis of the regional distribution by (-)-3H-dihydroalprenolol binding. *J. Cardiovasc. Pharmacol.* 4 34–43. 10.1097/00005344-198201000-00007 6176797

[B16] Bryant S. M. Kong C. H. T. Watson J. J. Gadeberg H. C. Roth D. M. Patel H. H. (2018). Caveolin-3 KO disrupts t-tubule structure and decreases t-tubular ICa density in mouse ventricular myocytes. *Am. J. Physiol. Heart Circ. Physiol.* 315 H1101–H1111. 10.1152/ajpheart.00209.2018 30028203 PMC6415741

[B17] Calaghan S. Kozera L. White E. (2008). Compartmentalisation of cAMP-dependent signalling by caveolae in the adult cardiac myocyte. *J. Mol. Cell. Cardiol.* 45 88–92. 10.1016/j.yjmcc.2008.04.004 18514221

[B18] Camors E. Charue D. Trouve P. Monceau V. Loyer X. Russo-Marie F. (2006). Association of annexin A5 with Na^+^/Ca^2+^ exchanger and caveolin-3 in non-failing and failing human heart. *J. Mol. Cell. Cardiol.* 40 47–55. 10.1016/j.yjmcc.2005.08.009 16330044

[B19] Campostrini G. Bonzanni M. Lissoni A. Bazzini C. Milanesi R. Vezzoli E. (2017). The expression of the rare caveolin-3 variant T78M alters cardiac ion channels function and membrane excitability. *Cardiovasc. Res.* 113 1256–1265. 10.1093/cvr/cvx122 28898996 PMC5852518

[B20] Cavalli A. Eghbali M. Minosyan T. Y. Stefani E. Philipson K. D. (2007). Localization of sarcolemmal proteins to lipid rafts in the myocardium. *Cell Calcium* 42 313–322. 10.1016/j.ceca.2007.01.003 17320949 PMC2724266

[B21] Chen C. C. Lamping K. G. Nuno D. W. Barresi R. Prouty S. J. Lavoie J. L. (2003). Abnormal coronary function in mice deficient in alpha1H T-type Ca2+ channels. *Science* 302 1416–1418. 10.1126/science.1089268 14631046

[B22] Cheng E. P. Yuan C. Navedo M. F. Dixon R. E. Nieves-Cintron M. Scott J. D. (2011). Restoration of normal L-type Ca^2+^ channel function during Timothy syndrome by ablation of an anchoring protein. *Circ. Res.* 109 255–261. 10.1161/CIRCRESAHA.111.248252 21700933 PMC3151468

[B23] Chiang D. Y. Li N. Wang Q. Alsina K. M. Quick A. P. Reynolds J. O. (2014). Impaired local regulation of ryanodine receptor type 2 by protein phosphatase 1 promotes atrial fibrillation. *Cardiovasc. Res.* 103 178–187. 10.1093/cvr/cvu123 24812280 PMC4133595

[B24] Christel C. J. Cardona N. Mesirca P. Herrmann S. Hofmann F. Striessnig J. (2012). Distinct localization and modulation of Cav1.2 and Cav1.3 L-type Ca^2+^ channels in mouse sinoatrial node. *J. Physiol.* 590 6327–6342. 10.1113/jphysiol.2012.239954 23045342 PMC3533195

[B25] Csepe T. A. Zhao J. Hansen B. J. Li N. Sul L. V. Lim P. (2016). Human sinoatrial node structure: 3D microanatomy of sinoatrial conduction pathways. *Prog. Biophys. Mol. Biol.* 120 164–178. 10.1016/j.pbiomolbio.2015.12.011 26743207 PMC4808362

[B26] Cunha S. R. Hund T. J. Hashemi S. Voigt N. Li N. Wright P. (2011). Defects in ankyrin-based membrane protein targeting pathways underlie atrial fibrillation. *Circulation* 124 1212–1222. 10.1161/CIRCULATIONAHA.111.023986 21859974 PMC3211046

[B27] Curran J. Musa H. Kline C. F. Makara M. A. Little S. C. Higgins J. D. (2015). Eps15 homology domain-containing protein 3 regulates cardiac T-type Ca^2+^ channel targeting and function in the atria. *J. Biol. Chem.* 290 12210–12221. 10.1074/jbc.M115.646893 25825486 PMC4424353

[B28] Delos Santos R. C. Garay C. Antonescu C. N. (2015). Charming neighborhoods on the cell surface: plasma membrane microdomains regulate receptor tyrosine kinase signaling. *Cell Signal.* 27 1963–1976. 10.1016/j.cellsig.2015.07.004 26163824

[B29] DiFrancesco D. (1993). Pacemaker mechanisms in cardiac tissue. *Annu. Rev. Physiol.* 55 455–472. 10.1146/annurev.ph.55.030193.002323 7682045

[B30] DiFrancesco D. Tortora P. (1991). Direct activation of cardiac pacemaker channels by intracellular cyclic AMP. *Nature* 351 145–147. 10.1038/351145a0 1709448

[B31] Dries E. Bito V. Lenaerts I. Antoons G. Sipido K. R. Macquaide N. (2013). Selective modulation of coupled ryanodine receptors during microdomain activation of calcium/calmodulin-dependent kinase II in the dyadic cleft. *Circ. Res.* 113 1242–1252. 10.1161/CIRCRESAHA.113.301896 24081880

[B32] Emery A. E. Dreifuss F. E. (1966). Unusual type of benign x-linked muscular dystrophy. *J. Neurol. Neurosurg. Psychiatry* 29 338–342. 10.1136/jnnp.29.4.338 5969090 PMC1064196

[B33] Fedorov V. V. Chang R. Glukhov A. V. Kostecki G. Janks D. Schuessler R. B. (2010). Complex interactions between the sinoatrial node and atrium during reentrant arrhythmias in the canine heart. *Circulation* 122 782–789. 10.1161/CIRCULATIONAHA.109.935288 20697021 PMC3001400

[B34] Fedorov V. V. Glukhov A. V. Chang R. (2012). Conduction barriers and pathways of the sinoatrial pacemaker complex: their role in normal rhythm and atrial arrhythmias. *Am. J. Physiol. Heart Circ. Physiol.* 302 H1773–H1783. 10.1152/ajpheart.00892.2011 22268110

[B35] Feron O. Smith T. W. Michel T. Kelly R. A. (1997). Dynamic targeting of the agonist-stimulated m2 muscarinic acetylcholine receptor to caveolae in cardiac myocytes. *J. Biol. Chem.* 272 17744–17748. 10.1074/jbc.272.28.17744 9211926

[B36] Foell J. D. Balijepalli R. C. Delisle B. P. Yunker A. M. Robia S. L. Walker J. W. (2004). Molecular heterogeneity of calcium channel beta-subunits in canine and human heart: evidence for differential subcellular localization. *Physiol. Genomics* 17 183–200. 10.1152/physiolgenomics.00207.2003 14762176

[B37] Froese A. Breher S. S. Waldeyer C. Schindler R. F. Nikolaev V. O. Rinne S. (2012). Popeye domain containing proteins are essential for stress-mediated modulation of cardiac pacemaking in mice. *J. Clin. Invest.* 122 1119–1130. 10.1172/JCI59410 22354168 PMC3287222

[B38] Galindo-Tovar A. Kaumann A. J. (2008). Phosphodiesterase-4 blunts inotropism and arrhythmias but not sinoatrial tachycardia of (-)-adrenaline mediated through mouse cardiac beta(1)-adrenoceptors. *Br. J. Pharmacol.* 153 710–720. 10.1038/sj.bjp.0707631 18084319 PMC2259196

[B39] Gao Z. Chen B. Mei-ling A. J. Wu Y. Guan X. Koval O. M. (2010). If and SR Ca^2+^ release both contribute to pacemaker activity in canine sinoatrial node cells. *J. Mol. Cell. Cardiol.* 49 33–40. 10.1016/j.yjmcc.2010.03.019 20380837 PMC2883640

[B40] Glukhov A. V. Balycheva M. Sanchez-Alonso J. L. Ilkan Z. Alvarez-Laviada A. Bhogal N. (2015a). Direct evidence for microdomain-specific localization and remodeling of functional L-type calcium channels in rat and human atrial myocytes. *Circulation* 132 2372–2384. 10.1161/CIRCULATIONAHA.115.018131 26450916 PMC4689179

[B41] Glukhov A. V. Kalyanasundaram A. Lou Q. Hage L. T. Hansen B. J. Belevych A. E. (2015b). Calsequestrin 2 deletion causes sinoatrial node dysfunction and atrial arrhythmias associated with altered sarcoplasmic reticulum calcium cycling and degenerative fibrosis within the mouse atrial pacemaker complex1. *Eur. Heart J.* 36 686–697. 10.1093/eurheartj/eht452 24216388 PMC4359358

[B42] Glukhov A. V. Fedorov V. V. Anderson M. E. Mohler P. J. Efimov I. R. (2010). Functional anatomy of the murine sinus node: high-resolution optical mapping of ankyrin-B heterozygous mice. *Am. J. Physiol. Heart Circ. Physiol.* 299 H482–H491. 10.1152/ajpheart.00756.2009 20525877 PMC2930390

[B43] Glukhov A. V. Hage L. T. Hansen B. J. Pedraza-Toscano A. Vargas-Pinto P. Hamlin R. L. (2013). Sinoatrial node reentry in a canine chronic left ventricular infarct model: role of intranodal fibrosis and heterogeneity of refractoriness. *Circ. Arrhythm. Electrophysiol.* 6 984–994. 10.1161/CIRCEP.113.000404 23960214 PMC3904863

[B44] Grimm M. Brown J. H. (2010). Beta-adrenergic receptor signaling in the heart: role of CaMKII. *J. Mol. Cell. Cardiol.* 48 322–330. 10.1016/j.yjmcc.2009.10.016 19883653 PMC2896283

[B45] Gu H. Neel B. G. (2003). The “Gab” in signal transduction. *Trends Cell Biol.* 13 122–130. 10.1016/S0962-8924(03)00002-3 12628344

[B46] Guo Y. Golebiewska U. Scarlata S. (2011). Modulation of Ca^2+^ activity in cardiomyocytes through caveolae-Gαq interactions. *Biophys. J.* 100 1599–1607. 10.1016/j.bpj.2011.02.013 21463572 PMC3072606

[B47] Hagiwara N. Irisawa H. Kameyama M. (1988). Contribution of two types of calcium currents to the pacemaker potentials of rabbit sino-atrial node cells. *J. Physiol.* 395 233–253. 10.1113/jphysiol.1988.sp016916 2457676 PMC1191991

[B48] Hohendanner F. Walther S. Maxwell J. T. Kettlewell S. Awad S. Smith G. L. (2015). Inositol-1,4,5-trisphosphate induced Ca^2+^ release and excitation-contraction coupling in atrial myocytes from normal and failing hearts. *J. Physiol.* 593 1459–1477. 10.1113/jphysiol.2014.283226 25416623 PMC4376424

[B49] Holm H. Gudbjartsson D. F. Sulem P. Masson G. Helgadottir H. T. Zanon C. (2011). A rare variant in MYH6 is associated with high risk of sick sinus syndrome. *Nat. Genet.* 43 316–320. 10.1038/ng.781 21378987 PMC3066272

[B50] Hoorntje E. T. Bollen I. A. Barge-Schaapveld D. Q. van Tienen F. H. Te Meerman G. J. Jansweijer J. A. (2017). Lamin A/C-related cardiac disease: late onset with a variable and mild phenotype in a large cohort of patients with the lamin A/C p.(Arg331Gln) founder mutation. *Circ. Cardiovasc. Genet.* 10:e001631. 10.1161/CIRCGENETICS.116.001631 28790152

[B51] Hua R. Adamczyk A. Robbins C. Ray G. Rose R. A. (2012). Distinct patterns of constitutive phosphodiesterase activity in mouse sinoatrial node and atrial myocardium. *PLoS One* 7:e47652. 10.1371/journal.pone.0047652 23077656 PMC3471891

[B52] Iancu R. V. Jones S. W. Harvey R. D. (2007). Compartmentation of cAMP signaling in cardiac myocytes: a computational study. *Biophys. J.* 92 3317–3331. 10.1529/biophysj.106.095356 17293406 PMC1852367

[B53] Jaleel N. Nakayama H. Chen X. Kubo H. MacDonnell S. Zhang H. (2008). Ca^2+^ influx through T- and L-type Ca^2+^ channels have different effects on myocyte contractility and induce unique cardiac phenotypes. *Circ. Res.* 103 1109–1119. 10.1161/CIRCRESAHA.108.185611 18832749 PMC2678411

[B54] Jones L. R. Suzuki Y. J. Wang W. Kobayashi Y. M. Ramesh V. Franzini-Armstrong C. (1998). Regulation of Ca^2+^ signaling in transgenic mouse cardiac myocytes overexpressing calsequestrin. *J. Clin. Invest.* 101 1385–1393. 10.1172/JCI1362 9525981 PMC508716

[B55] Jones S. A. Yamamoto M. Tellez J. O. Billeter R. Boyett M. R. Honjo H. (2008). Distinguishing properties of cells from the myocardial sleeves of the pulmonary veins: a comparison of normal and abnormal pacemakers. *Circ. Arrhythm. Electrophysiol.* 1 39–48. 10.1161/CIRCEP.107.748467 19808392

[B56] Ju Y. K. Liu J. Lee B. H. Lai D. Woodcock E. A. Lei M. (2011). Distribution and functional role of inositol 1,4,5-trisphosphate receptors in mouse sinoatrial node. *Circ. Res.* 109 848–857. 10.1161/CIRCRESAHA.111.243824 21852551

[B57] Karst M. L. Herron K. J. Olson T. M. (2008). X-linked nonsyndromic sinus node dysfunction and atrial fibrillation caused by emerin mutation. *J. Cardiovasc. Electrophysiol.* 19 510–515. 10.1111/j.1540-8167.2007.01081.x 18266676 PMC2367157

[B58] Kim M. S. Maltsev A. V. Monfredi O. Maltseva L. A. Wirth A. Florio M. C. (2018). Heterogeneity of calcium clock functions in dormant, dysrhythmically and rhythmically firing single pacemaker cells isolated from SA node. *Cell Calcium* 74 168–179. 10.1016/j.ceca.2018.07.002 30092494 PMC6402562

[B59] Knollmann B. C. Chopra N. Hlaing T. Akin B. Yang T. Ettensohn K. (2006). Casq2 deletion causes sarcoplasmic reticulum volume increase, premature Ca^2+^ release, and catecholaminergic polymorphic ventricular tachycardia. *J. Clin. Invest.* 116 2510–2520. 10.1172/JCI29128 16932808 PMC1551934

[B60] Lakatta E. G. DiFrancesco D. (2009). What keeps us ticking: a funny current, a calcium clock, or both? *J. Mol. Cell. Cardiol.* 47 157–170. 10.1016/j.yjmcc.2009.03.022 19361514 PMC4554526

[B61] Lakatta E. G. Maltsev V. A. Vinogradova T. M. (2010). A coupled SYSTEM of intracellular Ca^2+^ clocks and surface membrane voltage clocks controls the timekeeping mechanism of the heart’s pacemaker. *Circ. Res.* 106 659–673. 10.1161/CIRCRESAHA.109.206078 20203315 PMC2837285

[B62] Lang D. Warden A. Balijepalli R. Kamp T. J. Glukhov A. V. (2016). Loss of caveolin-3 disrupts mouse sinoatrial node pacemaking and stimulates atrial arrhythmogenesis. *Circulation* 134(Suppl 1), A15361.

[B63] Le Scouarnec S. Bhasin N. Vieyres C. Hund T. J. Cunha S. R. Koval O. (2008). Dysfunction in ankyrin-B-dependent ion channel and transporter targeting causes human sinus node disease. *Proc. Natl. Acad. Sci. U.S.A.* 105 15617–15622. 10.1073/pnas.0805500105 18832177 PMC2563133

[B64] Li N. Csepe T. A. Hansen B. J. Dobrzynski H. Higgins R. S. Kilic A. (2015). Molecular mapping of sinoatrial node HCN channel expression in the human heart. *Circ. Arrhythm. Electrophysiol.* 8 1219–1227. 10.1161/CIRCEP.115.003070 26304511 PMC4618238

[B65] Li N. Hansen B. J. Csepe T. A. Zhao J. Ignozzi A. J. Sul L. V. (2017). Redundant and diverse intranodal pacemakers and conduction pathways protect the human sinoatrial node from failure. *Sci. Transl. Med.* 9:eaam5607. 10.1126/scitranslmed.aam5607 28747516 PMC5775890

[B66] Li Y. Sirenko S. Riordon D. R. Yang D. Spurgeon H. Lakatta E. G. (2016). CaMKII-dependent phosphorylation regulates basal cardiac pacemaker function via modulation of local Ca^2+^ releases. *Am. J. Physiol. Heart Circ. Physiol.* 311 H532–H544. 10.1152/ajpheart.00765.2015 27402669 PMC5142178

[B67] Liao Z. Lockhead D. Larson E. D. Proenza C. (2010). Phosphorylation and modulation of hyperpolarization-activated HCN4 channels by protein kinase A in the mouse sinoatrial node. *J. Gen. Physiol.* 136 247–258. 10.1085/jgp.201010488 20713547 PMC2931151

[B68] Logantha S. J. Stokke M. K. Atkinson A. J. Kharche S. R. Parveen S. Saeed Y. (2016). Ca(2+)-clock-dependent pacemaking in the sinus node is impaired in mice with a cardiac specific reduction in SERCA2 abundance. *Front. Physiol.* 7:197. 10.3389/fphys.2016.00197 27313537 PMC4889599

[B69] Lu R. Chen Y. Cottingham C. Peng N. Jiao K. Limbird L. E. (2010). Enhanced hypotensive, bradycardic, and hypnotic responses to alpha2-adrenergic agonists in spinophilin-null mice are accompanied by increased G protein coupling to the alpha2A-adrenergic receptor. *Mol. Pharmacol.* 78 279–286. 10.1124/mol.110.065300 20430865 PMC2917858

[B70] Lukyanenko Y. O. Younes A. Lyashkov A. E. Tarasov K. V. Riordon D. R. Lee J. (2016). Ca^2+^/calmodulin-activated phosphodiesterase 1A is highly expressed in rabbit cardiac sinoatrial nodal cells and regulates pacemaker function. *J. Mol. Cell. Cardiol.* 98 73–82. 10.1016/j.yjmcc.2016.06.064 27363295 PMC5054686

[B71] Luo M. Guan X. Luczak E. D. Lang D. Kutschke W. Gao Z. (2013). Diabetes increases mortality after myocardial infarction by oxidizing CaMKII. *J. Clin. Invest.* 123 1262–1274. 10.1172/JCI65268 23426181 PMC3673230

[B72] Lyashkov A. E. Juhaszova M. Dobrzynski H. Vinogradova T. M. Maltsev V. A. Juhasz O. (2007). Calcium cycling protein density and functional importance to automaticity of isolated sinoatrial nodal cells are independent of cell size. *Circ. Res.* 100 1723–1731. 10.1161/CIRCRESAHA.107.153676 17525366

[B73] Macdougall D. A. Agarwal S. R. Stopford E. A. Chu H. Collins J. A. Longster A. L. (2012). Caveolae compartmentalise beta2-adrenoceptor signals by curtailing cAMP production and maintaining phosphatase activity in the sarcoplasmic reticulum of the adult ventricular myocyte. *J. Mol. Cell. Cardiol.* 52 388–400. 10.1016/j.yjmcc.2011.06.014 21740911 PMC3270222

[B74] Mangoni M. E. Couette B. Bourinet E. Platzer J. Reimer D. Striessnig J. (2003). Functional role of L-type Cav1.3 Ca^2+^ channels in cardiac pacemaker activity. *Proc. Natl. Acad. Sci. U.S.A.* 100 5543–5548. 10.1073/pnas.0935295100 12700358 PMC154381

[B75] Mangoni M. E. Traboulsie A. Leoni A. L. Couette B. Marger L. Le Quang K. (2006). Bradycardia and slowing of the atrioventricular conduction in mice lacking CaV3.1/alpha1G T-type calcium channels. *Circ. Res.* 98 1422–1430. 10.1161/01.RES.0000225862.14314.49 16690884

[B76] Marionneau C. Couette B. Liu J. Li H. Mangoni M. E. Nargeot J. (2005). Specific pattern of ionic channel gene expression associated with pacemaker activity in the mouse heart. *J. Physiol.* 562(Pt 1), 223–234. 10.1113/jphysiol.2004.074047 15498808 PMC1665484

[B77] Markandeya Y. S. Fahey J. M. Pluteanu F. Cribbs L. L. Balijepalli R. C. (2011). Caveolin-3 regulates protein kinase A modulation of the Ca(V)3.2 (alpha1H) T-type Ca^2+^ channels. *J. Biol. Chem.* 286 2433–2444. 10.1074/jbc.M110.182550 21084288 PMC3024737

[B78] Masson-Pevet M. Gros D. Besselsen E. (1980). The caveolae in rabbit sinus node and atrium. *Cell Tissue Res.* 208 183–196. 10.1007/BF00234869 7407830

[B79] Mohler P. J. Splawski I. Napolitano C. Bottelli G. Sharpe L. Timothy K. (2004). A cardiac arrhythmia syndrome caused by loss of ankyrin-B function. *Proc. Natl. Acad. Sci. U.S.A.* 101 9137–9142. 10.1073/pnas.0402546101 15178757 PMC428486

[B80] Mohler P. J. Schott J. J. Gramolini A. O. Dilly K. W. Guatimosim S. duBell W. H. (2003). Ankyrin-B mutation causes type 4 long-QT cardiac arrhythmia and sudden cardiac death. *Nature* 421 634–639. 10.1038/nature01335 12571597

[B81] Monfredi O. Tsutsui K. Ziman B. Stern M. D. Lakatta E. G. Maltsev V. A. (2018). Electrophysiological heterogeneity of pacemaker cells in the rabbit intercaval region, including the SA node: insights from recording multiple ion currents in each cell. *Am. J. Physiol. Heart Circ. Physiol.* 314 H403–H414. 10.1152/ajpheart.00253.2016 28916636 PMC5899256

[B82] Musa H. Lei M. Honjo H. Jones S. A. Dobrzynski H. Lancaster M. K. (2002). Heterogeneous expression of Ca^2+^ handling proteins in rabbit sinoatrial node. *J. Histochem. Cytochem.* 50 311–324. 10.1177/002215540205000303 11850434

[B83] Nichols C. B. Rossow C. F. Navedo M. F. Westenbroek R. E. Catterall W. A. Santana L. F. (2010). Sympathetic stimulation of adult cardiomyocytes requires association of AKAP5 with a subpopulation of L-type calcium channels. *Circ. Res.* 107 747–756. 10.1161/CIRCRESAHA.109.216127 20671242 PMC2981172

[B84] Nikolaev V. O. Moshkov A. Lyon A. R. Miragoli M. Novak P. Paur H. (2010). Beta2-adrenergic receptor redistribution in heart failure changes cAMP compartmentation. *Science* 327 1653–1657. 10.1126/science.1185988 20185685

[B85] Petzhold D. da Costa-Goncalves A. C. Gross V. Morano I. (2011). Spinophilin is required for normal morphology, Ca^2+^ homeostasis and contraction but dispensable for beta-adrenergic stimulation of adult cardiomyocytes. *J. Muscle Res. Cell Motil.* 32 243–248. 10.1007/s10974-011-9259-4 21922228

[B86] Protas L. Robinson R. B. (2000). Mibefradil, an I(Ca,T) blocker, effectively blocks I(Ca,L) in rabbit sinus node cells. *Eur. J. Pharmacol.* 401 27–30. 10.1016/S0014-2999(00)00364-2 10915833

[B87] Pulli I. Blom T. Lof C. Magnusson M. Rimessi A. Pinton P. (2015). A novel chimeric aequorin fused with caveolin-1 reveals a sphingosine kinase 1-regulated Ca^2+^ microdomain in the caveolar compartment. *Biochim. Biophys. Acta* 1853 2173–2182. 10.1016/j.bbamcr.2015.04.005 25892494

[B88] Ragusa M. J. Dancheck B. Critton D. A. Nairn A. C. Page R. Peti W. (2010). Spinophilin directs protein phosphatase 1 specificity by blocking substrate binding sites. *Nat. Struct. Mol. Biol.* 17 459–464. 10.1038/nsmb.1786 20305656 PMC2924587

[B89] Rigg L. Heath B. M. Cui Y. Terrar D. A. (2000). Localisation and functional significance of ryanodine receptors during beta-adrenoceptor stimulation in the guinea-pig sino-atrial node. *Cardiovasc. Res.* 48 254–264. 10.1016/S0008-6363(00)00153-X 11054472

[B90] Rybin V. O. Xu X. Lisanti M. P. Steinberg S. F. (2000). Differential targeting of beta -adrenergic receptor subtypes and adenylyl cyclase to cardiomyocyte caveolae. A mechanism to functionally regulate the cAMP signaling pathway. *J. Biol. Chem.* 275 41447–41457. 10.1074/jbc.M006951200 11006286

[B91] Scriven D. R. Klimek A. Asghari P. Bellve K. Moore E. D. (2005). Caveolin-3 is adjacent to a group of extradyadic ryanodine receptors. *Biophys. J.* 89 1893–1901. 10.1529/biophysj.105.064212 15980179 PMC1366692

[B92] Senzaki H. Smith C. J. Juang G. J. Isoda T. Mayer S. P. Ohler A. (2001). Cardiac phosphodiesterase 5 (cGMP-specific) modulates beta-adrenergic signaling in vivo and is down-regulated in heart failure. *FASEB J.* 15 1718–1726. 10.1096/fj.00-0538com 11481219

[B93] Shcherbakova O. G. Hurt C. M. Xiang Y. Dell’Acqua M. L. Zhang Q. Tsien R. W. (2007). Organization of beta-adrenoceptor signaling compartments by sympathetic innervation of cardiac myocytes. *J. Cell Biol.* 176 521–533. 10.1083/jcb.200604167 17296797 PMC2063986

[B94] Sprenger J. U. Perera R. K. Steinbrecher J. H. Lehnart S. E. Maier L. S. Hasenfuss G. (2015). In vivo model with targeted cAMP biosensor reveals changes in receptor-microdomain communication in cardiac disease. *Nat. Commun.* 6:6965. 10.1038/ncomms7965 25917898

[B95] St Clair J. R. Larson E. D. Sharpe E. J. Liao Z. Proenza C. (2017). Phosphodiesterases 3 and 4 differentially regulate the funny current, if, in mouse sinoatrial node myocytes. *J. Cardiovasc. Dev. Dis.* 4:10. 10.3390/jcdd4030010 28868308 PMC5573264

[B96] St Clair J. R. Liao Z. Larson E. D. Proenza C. (2013). PKA-independent activation of I(f) by cAMP in mouse sinoatrial myocytes. *Channels* 7 318–321. 10.4161/chan.25293 23756695 PMC3989360

[B97] Stern M. D. Maltseva L. A. Juhaszova M. Sollott S. J. Lakatta E. G. Maltsev V. A. (2014). Hierarchical clustering of ryanodine receptors enables emergence of a calcium clock in sinoatrial node cells. *J. Gen. Physiol.* 143 577–604. 10.1085/jgp.201311123 24778430 PMC4003189

[B98] Tingley W. G. Pawlikowska L. Zaroff J. G. Kim T. Nguyen T. Young S. G. (2007). Gene-trapped mouse embryonic stem cell-derived cardiac myocytes and human genetics implicate AKAP10 in heart rhythm regulation. *Proc. Natl. Acad. Sci. U.S.A.* 104 8461–8466. 10.1073/pnas.0610393104 17485678 PMC1866184

[B99] Tonegawa K. Otsuka W. Kumagai S. Matsunami S. Hayamizu N. Tanaka S. (2017). Caveolae-specific activation loop between CaMKII and L-type Ca^2+^ channel aggravates cardiac hypertrophy in alpha1-adrenergic stimulation. *Am. J. Physiol. Heart Circ. Physiol.* 312 H501–H514. 10.1152/ajpheart.00601.2016 28039202

[B100] Torrente A. G. Mesirca P. Neco P. Rizzetto R. Dubel S. Barrere C. (2016). L-type Cav1.3 channels regulate ryanodine receptor-dependent Ca^2+^ release during sino-atrial node pacemaker activity. *Cardiovasc. Res.* 109 451–461. 10.1093/cvr/cvw006 26786159

[B101] Torrente A. G. Zhang R. Zaini A. Giani J. F. Kang J. Lamp S. T. (2015). Burst pacemaker activity of the sinoatrial node in sodium-calcium exchanger knockout mice. *Proc. Natl. Acad. Sci. U.S.A.* 112 9769–9774. 10.1073/pnas.1505670112 26195795 PMC4534240

[B102] Tsui J. Inagaki M. Schulman H. (2005). Calcium/calmodulin-dependent protein kinase II (CaMKII) localization acts in concert with substrate targeting to create spatial restriction for phosphorylation. *J. Biol. Chem.* 280 9210–9216. 10.1074/jbc.M407653200 15582994

[B103] Tsutsui K. Monfredi O. J. Sirenko-Tagirova S. G. Maltseva L. A. Bychkov R. Kim M. S. (2018). A coupled-clock system drives the automaticity of human sinoatrial nodal pacemaker cells. *Sci. Signal.* 11:eaa7608 10.1126/scisignal.aap7608 PMC6138244 29895616

[B104] Unudurthi S. D. Wu X. Qian L. Amari F. Onal B. Li N. (2016). Two-pore K^+^ channel TREK-1 regulates sinoatrial node membrane excitability. *J. Am. Heart Assoc.* 5:e002865. 10.1161/JAHA.115.002865 27098968 PMC4859279

[B105] Vatta M. Ackerman M. J. Ye B. Makielski J. C. Ughanze E. E. Taylor E. W. (2006). Mutant caveolin-3 induces persistent late sodium current and is associated with long-QT syndrome. *Circulation* 114 2104–2112. 10.1161/CIRCULATIONAHA.106.635268 17060380

[B106] Vinogradova T. M. Kobrinsky E. Lakatta E. G. (2018). Dual activation of phosphodiesterases 3 and 4 regulates basal spontaneous beating rate of cardiac pacemaker cells: role of compartmentalization? *Front. Physiol.* 9:1301. 10.3389/fphys.2018.01301 30356755 PMC6189467

[B107] Vinogradova T. M. Lakatta E. G. (2009). Regulation of basal and reserve cardiac pacemaker function by interactions of cAMP-mediated PKA-dependent Ca^2+^ cycling with surface membrane channels. *J. Mol. Cell. Cardiol.* 47 456–474. 10.1016/j.yjmcc.2009.06.014 19573534 PMC2757791

[B108] Vinogradova T. M. Lyashkov A. E. Zhu W. Ruknudin A. M. Sirenko S. Yang D. (2006). High basal protein kinase A-dependent phosphorylation drives rhythmic internal Ca^2+^ store oscillations and spontaneous beating of cardiac pacemaker cells. *Circ. Res.* 98 505–514. 10.1161/01.RES.0000204575.94040.d1 16424365

[B109] Vinogradova T. M. Sirenko S. Lyashkov A. E. Younes A. Li Y. Zhu W. (2008). Constitutive phosphodiesterase activity restricts spontaneous beating rate of cardiac pacemaker cells by suppressing local Ca^2+^ releases. *Circ. Res.* 102 761–769. 10.1161/CIRCRESAHA.107.161679 18276917

[B110] Vinogradova T. M. Zhou Y. Y. Bogdanov K. Y. Yang D. Kuschel M. Cheng H. (2000). Sinoatrial node pacemaker activity requires Ca^2+^/calmodulin-dependent protein kinase II activation. *Circ. Res.* 87 760–767. 10.1161/01.RES.87.9.760 11055979

[B111] Vinogradova T. M. Zhou Y. Y. Maltsev V. Lyashkov A. Stern M. Lakatta E. G. (2004). Rhythmic ryanodine receptor Ca^2+^ releases during diastolic depolarization of sinoatrial pacemaker cells do not require membrane depolarization. *Circ. Res.* 94 802–809. 10.1161/01.RES.0000122045.55331.0F 14963011

[B112] Walden A. P. Dibb K. M. Trafford A. W. (2009). Differences in intracellular calcium homeostasis between atrial and ventricular myocytes. *J. Mol. Cell. Cardiol.* 46 463–473. 10.1016/j.yjmcc.2008.11.003 19059414

[B113] Warrier S. Belevych A. E. Ruse M. Eckert R. L. Zaccolo M. Pozzan T. (2005). Beta-adrenergic- and muscarinic receptor-induced changes in cAMP activity in adult cardiac myocytes detected with FRET-based biosensor. *Am. J. Physiol. Cell Physiol.* 289 C455–C461. 10.1152/ajpcell.00058.2005 15788489

[B114] Warrier S. Ramamurthy G. Eckert R. L. Nikolaev V. O. Lohse M. J. Harvey R. D. (2007). cAMP microdomains and L-type Ca^2+^ channel regulation in guinea-pig ventricular myocytes. *J. Physiol.* 580(Pt 3), 765–776. 10.1113/jphysiol.2006.124891 17289786 PMC2075464

[B115] Wehrens X. H. Lehnart S. E. Reiken S. R. Marks A. R. (2004). Ca^2+^/calmodulin-dependent protein kinase II phosphorylation regulates the cardiac ryanodine receptor. *Circ. Res.* 94 e61–e70. 10.1161/01.RES.0000125626.33738.E2 15016728

[B116] Whittaker C. A. Hynes R. O. (2002). Distribution and evolution of von Willebrand/integrin A domains: widely dispersed domains with roles in cell adhesion and elsewhere. *Mol. Biol. Cell* 13 3369–3387. 10.1091/mbc.e02-05-0259 12388743 PMC129952

[B117] Wright P. T. Nikolaev V. O. O’Hara T. Diakonov I. Bhargava A. Tokar S. (2014). Caveolin-3 regulates compartmentation of cardiomyocyte beta2-adrenergic receptor-mediated cAMP signaling. *J. Mol. Cell. Cardiol.* 67 38–48. 10.1016/j.yjmcc.2013.12.003 24345421 PMC4266930

[B118] Wu Y. Anderson M. E. (2014). CaMKII in sinoatrial node physiology and dysfunction. *Front. Pharmacol.* 5:48 10.3389/fphar.2014.00048 PMC3957193 24672485

[B119] Wu Y. Valdivia H. H. Wehrens X. H. Anderson M. E. (2016). A single protein kinase A or calmodulin kinase II site does not control the cardiac pacemaker Ca^2+^ clock. *Circ. Arrhythm. Electrophysiol.* 9:e003180. 10.1161/CIRCEP.115.003180 26857906 PMC4755317

[B120] Yanni J. Tellez J. O. Maczewski M. Mackiewicz U. Beresewicz A. Billeter R. (2011). Changes in ion channel gene expression underlying heart failure-induced sinoatrial node dysfunction. *Circ. Heart Fail.* 4 496–508. 10.1161/CIRCHEARTFAILURE.110.957647 21565973

[B121] Ye B. Balijepalli R. C. Foell J. D. Kroboth S. Ye Q. Luo Y. H. (2008). Caveolin-3 associates with and affects the function of hyperpolarization-activated cyclic nucleotide-gated channel 4. *Biochemistry* 47 12312–12318. 10.1021/bi8009295 19238754 PMC2803323

[B122] Younes A. Lyashkov A. E. Graham D. Sheydina A. Volkova M. V. Mitsak M. (2008). Ca^2+^-stimulated basal adenylyl cyclase activity localization in membrane lipid microdomains of cardiac sinoatrial nodal pacemaker cells. *J. Biol. Chem.* 283 14461–14468. 10.1074/jbc.M707540200 18356168 PMC2386925

[B123] Zaccolo M. Pozzan T. (2002). Discrete microdomains with high concentration of cAMP in stimulated rat neonatal cardiac myocytes. *Science* 295 1711–1715. 10.1126/science.1069982 11872839

[B124] Zhang Z. Xu Y. Song H. Rodriguez J. Tuteja D. Namkung Y. (2002). Functional roles of Ca(v)1.3 (alpha(1D)) calcium channel in sinoatrial nodes: insight gained using gene-targeted null mutant mice. *Circ. Res.* 90 981–987. 10.1161/01.RES.0000018003.14304.E2 12016264

